# Ellagitannin HeT obtained from strawberry leaves is oxidized by bacterial membranes and inhibits the respiratory chain

**DOI:** 10.1002/2211-5463.12361

**Published:** 2018-01-04

**Authors:** Gustavo G. Martos, Alicia Mamani, María P. Filippone, Pedro O. Abate, Néstor E. Katz, Atilio P. Castagnaro, Juan C. Díaz Ricci

**Affiliations:** ^1^ INSIBIO (CONICET‐UNT) and Instituto de Química Biológica San Miguel de Tucuman Argentina; ^2^ Sección Biotecnología de la Estación Experimental Agroindustrial Obispo Colombres (EEAOC) Tucumán Argentina; ^3^ INQUINOA (UNT‐CONICET) Facultad de Bioquímica Química y Farmacia Universidad Nacional de Tucumán Argentina

**Keywords:** antimicrobial, ellagitannin, membrane interaction, respiration

## Abstract

Plant secondary metabolism produces a variety of tannins that have a wide range of biological activities, including activation of plant defenses and antimicrobial, anti‐inflammatory and antitumoral effects. The ellagitannin HeT (1‐*O*‐galloyl‐2,3;4,6‐bis‐hexahydroxydiphenoyl‐β‐d‐glucopyranose) from strawberry leaves elicits a strong plant defense response, and exhibits antimicrobial activity associated to the inhibition of the oxygen consumption, but its mechanism of action is unknown. In this paper we investigate the influence of HeT on bacterial cell membrane integrity and its effect on respiration. A β‐galactosidase unmasking experiment showed that HeT does not disrupt membrane integrity. Raman spectroscopy analysis revealed that HeT strongly interacts with the cell membrane. Spectrochemical analysis indicated that HeT is oxidized in contact with bacterial cell membranes, and functional studies showed that HeT inhibits oxygen consumption, NADH and MTT reduction. These results provide evidence that HeT inhibits the respiratory chain.

**Abbreviations**
CFU: colony forming unit
DPPH•: 2,2‐di(4‐*tert*‐octylphenyl)‐1‐picrylhydrazyl radical
ET: ellagitannin
HeT: 1‐*O*‐galloyl‐2,3,4,6‐bis‐hexahydroxydiphenoyl‐β‐d‐glucopyranose
HeTox: oxidized HeT
HeTred: reduced HeT
MTT: 3‐(4,5‐dimethylthiazolyl‐2)‐2,5‐diphenyltetrazolium bromide
ONPG: *ortho*‐nitrophenyl‐β‐galactoside

Ellagitannins (ETs) constitute one of the major classes of polyphenols derived from the secondary metabolism of plants and exhibit a wide range of biological activities, such as antimicrobial, anti‐inflammatory and antitumoral, along with activation of plant defenses 1, 2, 3. These effects are mainly attributed to their anti‐ or pro‐oxidant activities 4, their capacity to alter the cellular redox balance, or to regulate the accumulation of reactive oxygen species 5. It was also reported that some ETs can cause cell membrane damage and cell lysis, and inhibit the growth of pathogenic bacteria and fungi 6, 7, 8, 9, 10. We have previously reported the isolation from strawberry leaves of a 936 Da ellagitannin (1‐*O*‐galloyl‐2,3;4,6‐bis‐hexahydroxydiphenoyl‐β‐d‐glucopyranose) called HeT 3. It was shown that HeT elicits from plants a strong defense response against anthracnose disease caused by the virulent isolate of the fungal pathogen *Colletotrichum acutatum* 3, and also exhibits a direct antimicrobial activity against other pathogens, as reported earlier for another 316 Da compound also obtained from strawberry leaves called fragarin 11. These authors showed that fragarin affected the respiration of the bacterium *Clavibacer michiganensis*, showing a bacteriolytic effect, and these effects were due to the alteration of the cellular membrane integrity 12. Since fragarin and HeT are obtained from strawberry leaves, and both elicit the plant defense response and have antimicrobial properties, we were interested in investigating whether the antimicrobial activity was also due to the disruption of membrane integrity, and whether the redox properties of HeT and its interaction with bacterial cell membrane are implicated.

In view of the fact that HeT may be used as an active agent for pharmaceutical products to control skin infections produced by Gram‐positive pathogens, it is essential to study the mechanisms that would explain the antibacterial activity.

## Materials and methods

### HeT extraction and purification

HeT was obtained and purified from leaves of strawberry (*Fragaria ananassa)* cv. Pájaro according to Mamani *et al*. 3.

### Microorganisms and media

For oxygen consumption experiments and membrane preparation, the strain C5 of *Clavibacter michiganensis* subsp. *Sepedonicus* was used (provided by A. Molina, ETSIA, Madrid, Spain), and cultured in Luria–Bertani (LB) broth at 28 °C for 12 h. The strain CRL35 (*lacZ* ^+^) of *Enterococcus mundtii* (provided by Centro de Referencia para Lactobacilos, Tucumán, Argentina) was cultured in LAPT‐lactose broth (peptone, 15 g·L^−1^; tryptone, 10 g·L^−1^; yeast extract, 10 g·L^−1^; lactose, 10 g·L^−1^; Tween‐80, 0.1% v/v) for 16 h at 30 °C.

### Viability evaluation

**Figure 1:**
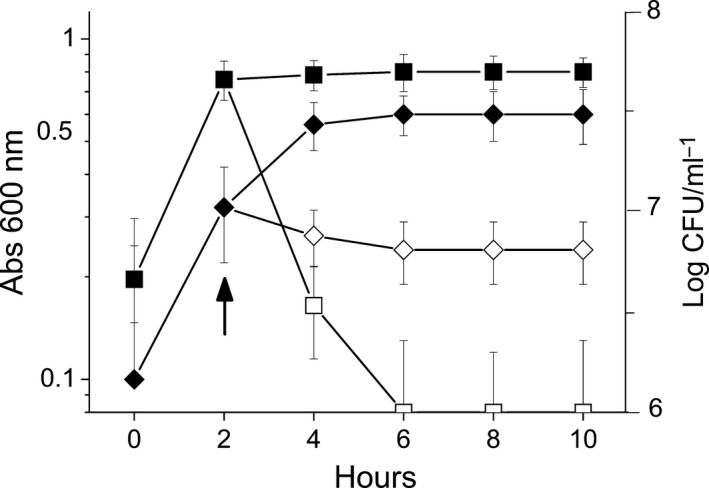
Effect of HeT on the growth (*A*_600_; ♦, ♢) and viability (CFU·mL‐1; ■, □) of *C. michiganensis*. The filled and open symbols correspond to cultures not treated or treated with HeT, respectively. The arrow indicates the moment when HeT (20 μm) was added. Results correspond to one of three identical experiments.

The effect of HeT on cell growth and viability was evaluated on *C. michiganensis* cultures (30 mL). Growth was followed by measuring the absorbance at 600 nm. The growth inhibition effect was tested by adding HeT (20 μm) to the liquid culture medium at the indicated time (see Fig. 1). Bacterial viability was evaluated by counting colonies (colony forming units (CFU)·mL^−1^) from suspension samples taken at different times, and plating on LB medium without HeT.

### Antiradical activity evaluation (evaluation of redox activity)

The antiradical activity of HeT was determined according to Burda and Oleszek 13 using an ethanolic solution of 2,2‐di(4‐*tert*‐octylphenyl)‐1‐picrylhydrazyl radical (DPPH•) measuring the decrease of absorbance at 515 nm at 30 min in a spectrophotometer (Beckman DU7500, Fullerton, CA, USA).

### β‐Galactosidase unmasking assay

**Figure 2:**
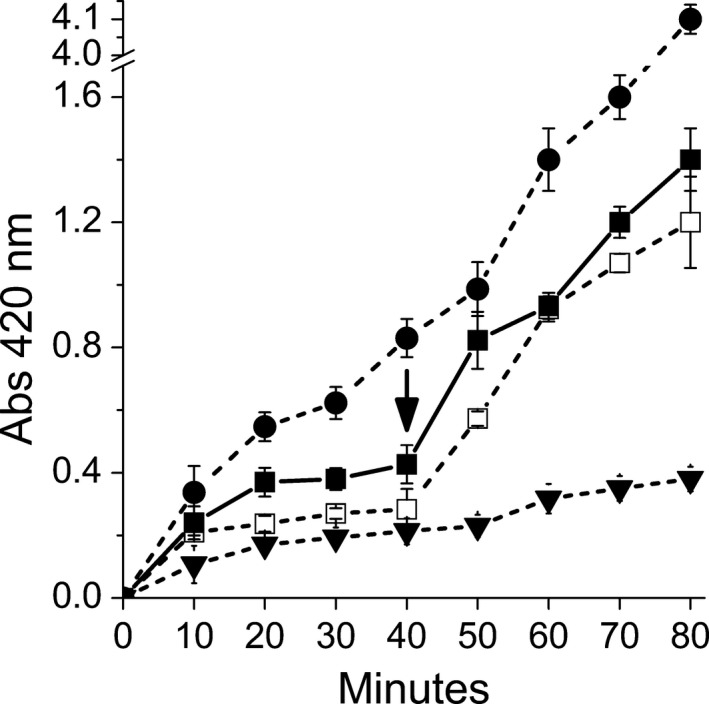
Effect of HeT on the permeabilization of cell membrane of *E. mundtii* CRL35. The permeabilization was detected by evaluating the β‐galactosidase activity at different times in the supernatant of cells suspended in ONPG‐phosphate buffer without HeT (**□**) or containing HeT (20 μm) (■); the arrow indicates the moment when SDS (0.1%) and chloroform was added to the suspension. Controls correspond to β‐gal activity detected in: (a) the supernatant of cells suspended in ONPG‐phosphate buffer with SDS (0.1%) and chloroform from the beginning (●), and (b) ONPG‐phosphate buffer supplemented with HeT (20 μm) free of cells (▼). Curves represent one example of three independent experiments. Abs, absorbance.

To evaluate the effect of HeT on cell membrane integrity, β‐galactosidase unmasking experiments were carried out with the strain CRL35 (*lacZ* ^+^) of *Enterococcus mundtii*. The latter is a Gram‐positive bacterium that produces an intracellular β‐ galactosidase, and hence its activity will be detected in the culture broth only if the cell membrane integrity is disrupted. Experiments consisted in detecting β‐galactosidase activity at different times in the supernatant of *E. mundtii* CRL35 suspensions that were treated with HeT or not. β‐Galactosidase activity was detected with *ortho*‐nitrophenyl‐β‐galactoside (ONPG) according to Miller 14. Briefly, *E. mundtii* CRL35 grown in LAPT‐lactose to induce the β‐galactosidase were collected at log‐phase (*A*_600_ = 0.3), centrifuged (5000 ***g***), washed once with phosphate buffer (100 mm, pH 7.6), resuspended up to a *D*_600_ = 1.0 in the same buffer containing ONPG (2 mm), and divided in two aliquots; one was supplemented with HeT (20 μm) and the other was without HeT (as control). Suspensions were then incubated at 28 °C with agitation (200 rpm). Samples of 0.5 mL were taken at different times and 0.25 mL of Na_2_CO_3_ added (1 m) to stop the reaction; they were centrifuged for 5 min at 12 000 ***g*** to remove the cell debris and the absorbance of the supernatant was measured at 420 nm. Control experiments were performed to verify the intracellular expression of the β‐galactosidase and to rule out any effect of HeT on ONPG (see Fig. 2).

### Membrane preparation

Cells of *C. michiganensis* were harvested from 100 mL culture and washed once with 20 mm phosphate buffer (pH 7.5). Washed cells were resuspended in the same buffer, disrupted by passing three times through a French press at 1200 kg·cm^−2^, and debris removed by centrifugation at 12 000 ***g*** (10 min at 4 °C). The cell‐free extract was ultracentrifuged at 100 000 ***g*** (1 h at 4 °C). The pellet containing the membranes was washed and resuspended in the same buffer 15. Protein was determined according to Lowry 16, using bovine serum albumin as standard.

### UV‐visible spectra

UV‐visible spectra of membrane suspensions (5 μg·mL^−1^), pure HeT (0.4 mm) and a mixture of both in phosphate buffer (pH 7.4) were acquired using a spectrophotometer (Beckman DU7500). The differential spectra among them were also obtained.

### Fourier transform infrared spectroscopy

Fourier transform infrared (FT‐IR) spectroscopy of HeT (50 μg) embedded into potassium bromide was obtained and recorded in the range 2000–600 cm^−1^, using a Perkin Elmer 1600 Series FT‐IR spectrometer (Waltham, MA, USA). Assignments of the major absorption bands were made with the IR program asia v. 2.0.

### Raman spectra

The Raman spectra of pure HeT (50 μm), cell membrane (3 mg·mL^−1^) and the mixture of HeT (50 μm) and membrane (3 mg·mL^−1^) in phosphate buffer (pH 7.4) were obtained. Raman spectra in the range 2000–600 cm^−1^ were obtained at room temperature with a solid state 532 nm green laser (10 mW), and slit aperture of 50 μm, using a Thermo Scientific DXR confocal Raman microscope (Madison, WI, USA) equipped with CCD detector. The resolution of the Raman shift was 4 cm^−1^, with a grating groove density of 900 lines·mm^−1^, and an accumulation rate of 60 spectra per sample.

### Differential pulse voltammetry and spectroelectrochemical measurements

HeT absorption spectra were recorded on a Varian Cary 50 spectrophotometer (Mulgrave, Australia), using 1 cm quartz cells. Electrochemistry experiments were carried out using BAS Epsilon EC Equipment (West Lafayette, IN, USA), with vitreous carbon as working electrode, Pt wire as auxiliary electrode, and Ag/AgCl (3 m KCl) as reference electrode. HeT solutions (0.4 mm) were prepared in phosphate buffer (pH 7.4). Spectroelectrochemistry in the UV‐visible range (300–800 nm) was carried out using a Honeycomb Cell from PINE Research Instrumentation (Durham, NC, USA). Oxidative difference spectra were acquired each 30 s at +0.3 V.

### Membrane redox activity

The effects of HeT (50 μm) on the redox membrane activities were determined using purified membranes of *C. michiganensis*. NADH consumption was measured at 340 nm (molar extinction coefficient of 6.22 mm ^−1^·cm^−1^), and expressed as micromoles of NADPH consumed per minute. The reduction of 3‐(4,5‐dimethylthiazol‐2‐yl)‐2,5‐diphenyltetrazolium bromide (MTT) to formazan was carried out with membranes pretreated with KCN, in the presence of NADH, measured at 570 nm (molar extinction coefficient of 16.9 mm ^−1^·cm^−1^), and expressed as millimoles of formazan produced per minute. Measurement were carried out at 37 °C in 0.5 mL using a spectrophotometer (Beckman DU7500) 17. KCN (5 mm) was used to inhibit electron transfer to oxygen.

### Oxygen consumption measurements

The oxygen consumption was measured on bacterial and membrane extract suspensions by respirometry using an Oroboros Oxygraph‐2k (Oroboros Instruments, Innsbruck, Austria) in a 2 mL chamber and low agitation (200 rpm) 18. HeT at different concentrations was added to the cuvette containing a culture of *C. michiganensis* grown in LB medium at log‐phase (*A*_600_ = 0.5) and 28 °C, and oxygen consumption online recorded. When purified membranes were used, HeT (50 μm) was added to the suspension (0.4 mg·mL^−1^) and measurements carried out at 37 °C. Controls corresponded to suspensions treated with KCN (5 mm).

### Statistical analyses

The statistical analyses of the data were carried out using infostat software (professional version 1.1, Córdoba, Argentina). All data were obtained from three independent experiments, and expressed as mean ± standard error. The data were also analyzed by one‐way analysis of variance (ANOVA), and the means were separated using Tukey's test with *P *<* *0.05.

## Results

### Effect of HeT on bacterial respiration

Experiments carried out with the bacterium *C. michiganensis* conducted to evaluate the effect of HeT on the oxygen consumption revealed a clear inhibition of the oxygen consumption rate, and the effect was dose dependent (Fig. S1).

### The effect of HeT on viability

With the aim of investigating how HeT affects bacterial growth, cultures of the bacterium *C. michiganensis* treated with HeT were evaluated. Results showed that the growth of *C. michiganensis* was halted immediately after the addition of HeT but, since the absorbance did not drop after HeT treatment (Fig. 1), we concluded that no lysis took place. The viability, evaluated as the capacity of bacteria to grow on a HeT‐free medium after the treatment with HeT, dropped immediately after the treatment (Fig. 1), indicating that HeT exerts a bactericide effect on the bacterium.

### Cell membrane integrity

With the aim of investigating whether the bactericidal effect was due to the disruption of cell membrane integrity, β‐galactosidase unmasking experiments were carried out with the strain CRL35 of *Enterococcus mundtii*, which expresses an intracellular β‐ galactosidase. Results showed that when *E. mundtii* CRL35 was in contact with HeT (20 μm), cells did not release the β‐galactosidase to the medium unless SDS and chloroform were added to permeabilize the membrane (Fig. 2). This outcome suggests that HeT affects respiration by a mechanism that does not involve the disruption of the cell membrane integrity, and confirms that HeT exerts no bacteriolytic effect on cells (Fig. 1).

### Spectral analysis of Raman scattering

**Figure 3:**
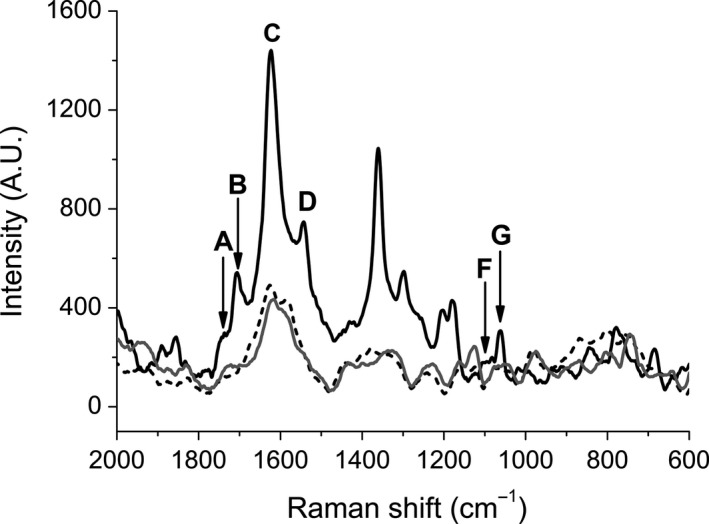
Raman spectra of HeT (solid line), membrane (dotted line) and the mixture of HeT and membrane (gray line). HeT (50 μm) and membrane were prepared in phosphate buffer (pH 7.4). Spectra correspond to averages of 60 spectra per sample. The letters correspond to the chemical groups assigned from the IR spectrum (see Fig. S2): A and B, carbonyls; C and D, aromatics; F and G, aromatic esters.

Raman spectra of pure HeT, purified bacterial membrane obtained from *C. michiganensis* and a mixture of both were analyzed. Previously, the infrared spectrum of pure HeT was obtained for band assignment (Fig. S2). Bands corresponding to carbonyls (A: 1737.2 cm^−1^; B: 1673.3 cm^−1^), aromatics (C: 1613.6 cm^−1^; D: 1518.7 cm^−1^), methylenes (E: 1446.7 cm^−1^) and aromatic esters (F: 1110.3 cm^−1^; G: 1078.2 cm^−1^) were identified and partially assigned to the Raman peaks (Fig. 3). Comparison of Raman spectra of HeT in buffer, with and without membrane fraction, showed that the signals corresponding to the bands assigned to carbonyl groups (A and B), aromatic groups (C and D) and aromatic esters (F and G) were completely quenched as result of the interaction with the membrane, suggesting that mainly the aromatic residues are involved in the interaction. This result indicates that HeT strongly interacts with the cell membrane.

### Electrochemical characterization of HeT

Experiments conducted to determine whether HeT exhibits redox activity showed that HeT is oxidized in the presence of DPPH• (Fig. S3). The latter suggests that HeT is partially reduced, and can transfer electrons to a suitable acceptor. Analysis of the redox potential of pure HeT determined by differential pulse voltammetry showed that HeT is oxidized at *V* = +0.164 V (Fig. S4). However, no detectable reduction potential was observed in the cyclic voltammetry (not shown) indicating that the oxidized form of HeT (HeTox) was stable under the conditions tested, and its formation was irreversible. Spectral analyses performed at +0.3 V revealed that the pure HeT exhibited significant changes as the degree of oxidation increased. Reduced HeT species (HeTred) exhibited no bands within the range of wavelengths analyzed (300–800 nm), whereas as HeT becomes more oxidized (HeTox), three clear bands emerged at λ_max_ = 365, 440 and 490 nm (Fig. S5).

### Interaction of HeT with bacterial cell membrane

**Figure 4:**
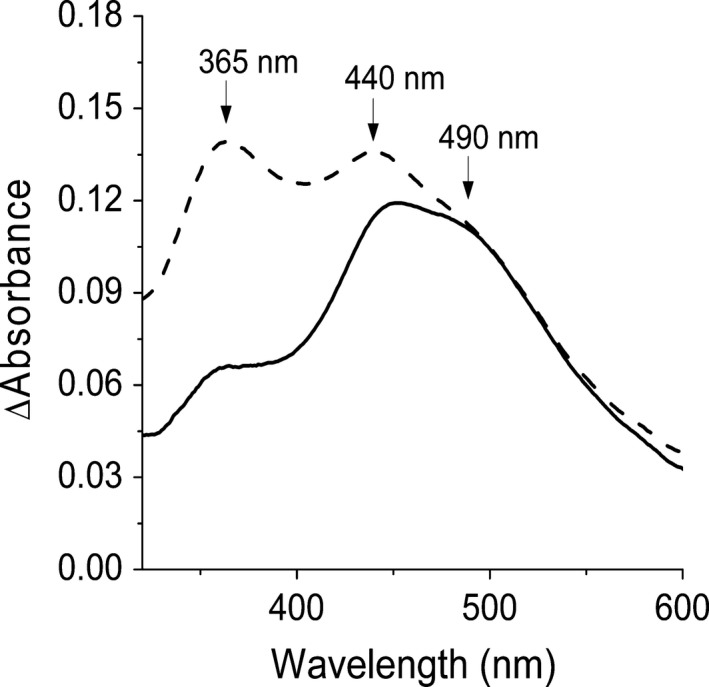
Differential UV‐visible spectra of HeT. The dotted line corresponds to the differential absorption spectrum between the completely reduced and the most oxidized HeT (0.4 mm) without membrane (taken from Fig. S5). The solid line corresponds to the differential absorption spectrum between a solution of HeT (0.4 mm) before and after membrane addition (5 μg·mL^−1^).

With the aim of investigating whether the bacterial membrane causes a modification of the HeT redox status, the differential spectrum of pure HeT in the presence of cell membrane was analyzed (Fig. 4). Previously the spectra of cell membrane and pure HeT, and the differential spectrum between them were obtained and analyzed. The latter showed that bacterial membrane does not contribute significantly to the spectrum of HeT when examined separately (Fig. S6). However, since HeT mixed with the membrane displayed a spectrum with three bands as HeTox (see Fig. S5), we can conclude that the presence of the bacterial membrane not only changes the HeT spectrum, indicating a strong interaction, as observed by Raman spectroscopy (Fig. 3), but that HeT also becomes oxidized. The difference of the peak intensities of pure HeTox and those of HeT in the presence of the membrane could be attributed to the different environment encountered by HeT when interacting with membrane components.

### Functional assays of the membrane

**Figure 5:**
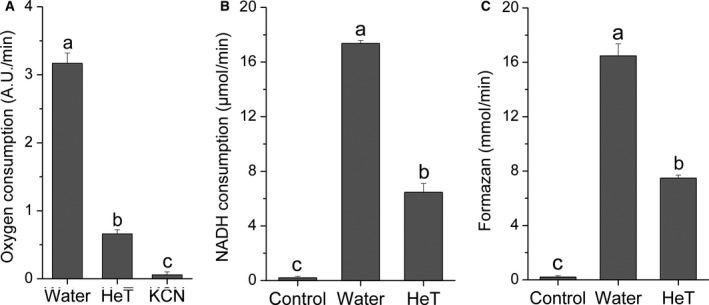
Oxygen consumption (A), NADH reduction (B) and formazan formation (C) of *C. michiganensis* membranes (0.4 mg·mL^−1^) treated with water, HeT (50 μm) or KCN (5 mm). Controls in (B,C) correspond to a solution of HeT or MTT without membrane. AU, arbitrary units. Values represent average of three repetitions. Error bars represent relative errors. Different letters represent statistically different values (Tukey's test, *P *<* *0.05).

Functional assays with purified membranes were performed to evaluate whether HeT causes the alteration of the membrane electron flux. Membranes treated with HeT showed a clear reduction of the rate of oxygen consumption (Fig. 5A), NADH consumption (Fig. 5B) and formazan formation from MTT (Fig. 5C). The direct interaction between NADH, MTT and HeT was ruled out by a control experiment carried out without membrane (Fig. 5B,C) indicating that HeT requires the membrane to exert its effect.

## Discussion

The antimicrobial activity of hydrolysable tannins, the interaction of ellagitannins with membranes, and their antibacterial activity associated to the alteration of the membrane potential have already been reported 6, 19, 20, 21, 22, 23.

In a previous paper it was shown that a low molecular mass compound called fragarin (316 Da), found in strawberry leaves, exhibited not only plant defense induction properties but also antimicrobial activity affecting oxygen consumption 11. These authors demonstrated that the inhibition of oxygen consumption was due to the disruption of cell membrane integrity, which caused a drop of the transmembrane potential and had a bacteriolytic effect on cell suspension 12. In contrast, β‐galactosidase unmasking experiments carried out with HeT indicated that this ellagitannin, although it also exhibits antimicrobial properties and causes the inhibition of the oxygen consumption, as reported for fragarin 11, 12, does not bring about cell membrane disruption; thus, the mechanism underlying the bactericidal effect is not the same. We hypothesized, therefore, that the mechanisms by which HeT affects respiration may be related to the interaction with cell membrane and its intrinsic redox properties. The first piece of evidence supporting the latter hypothesis was provided by Raman spectroscopy analyses revealing that HeT does interact with the membrane. The second piece of evidence came from HeT electrochemical analyses showing that HeT is oxidized by cell membrane, and the third piece of evidence came from the fact that HeT inhibits membrane NADH, MTT and oxygen consumption.

It has been demonstrated that the redox properties of tannins may change from anti‐ to pro‐oxidant as the oxygen partial pressure increases, producing spontaneous free radicals 24, 25 or working an electron scavenger 26, 27, 28. This information led us to speculate initially that HeT may function as an electron scavenger of the respiratory chain, as reported previously 29, 30. The latter was plausible since, from the reported structure of HeT, the number galloyl and hydroxyl groups exposed 3 led us to assume that it could function as a sink for the electrons needed for the respiration chain. However, since electrochemical experiments showed that HeT can only be oxidized, and that it is effectively oxidized in the presence of membrane, a new mechanism should be found. Perturbations of phospholipid membranes were reported for diterpenoidstotarol 31, abietic acid 32, resveratrol 33 and the galloyl group of catechins 34. The latter let us speculate that HeT tightly binds to the membrane, becomes oxidized and affects the electron flow through the membrane and hence the oxygen consumption. Nonetheless, we cannot completely rule out that HeT may also exert a direct inhibitory effect on the participating dehydrogenases. It could also be plausible that if the galloyl groups of HeT become inserted into the phospholipid palisade of the cell membrane, HeT would not only cause a change of the membrane structure or fluidity that could affect enzyme activity 35, but would also expose HeT to a totally new environment that may lead to the hydrolysis of any of the galloyl groups contained in HeT, as reported elsewhere 36. Further experiments are being conducted to elucidate possible mechanisms of interaction between HeT and membrane phospholipids that bring about the inhibition of the respiratory chain.

## Author contributions

Biological experiments were carried out by GGM and AM. Electrochemical experiments and data analysis were carried out by POA, NEK and GGM. The experimental design, data analysis and paper writing were carried out by JCDR, APC and GGM.

## Supporting information


**Fig. S1.** Oxygen consumption of *C. michiganensis* suspensions (*A*_600_ = 0.5) treated with water, increasing concentrations of HeT and KCN (5 mm). The values represent average of three repetitions. Error bars represent relative errors.
**Fig. S2.** Infrared spectrum of pure HeT (50 μm). Group assignments correspond to: carbonyls (A: 1737.2) and (B: 1673.3); aromatics (C, 1613.6) and (D, 1518.7); methylenes (E, 1446.7); aromatic esters (F, 1110.3) and (G, 1078.2).
**Fig. S3.** Effect of HeT on the reduction DPPH expressed as percentage of antiradical activity (%AA). Water was used as a control. The values represent means of three repetitions. Error bars represent relative errors.
**Fig. S4.** Differential pulse voltammetry of HeT (0.4 mm) in the oxidative range in aqueous phosphate buffer (pH 7.4).
**Fig. S5.** UV‐visible spectra of HeT with different level of oxidation. Oxidation was achieved by controlled potential electrolysis of HeT (0.4 mm) in phosphate buffer (pH 7.4) at +0.3 V. Spectra were acquired every 30 s.
**Fig. S6.** UV‐visible spectra of (A) bacterial membrane (5 μg·mL^−1^), (B) pure HeT (0.4 mm), and (C) HeT (0.4 mm) after reacting with the bacterial membrane (5 μg·mL^−1^). All solutions were prepared in phosphate buffer (pH 7.4).
