# Supplementary material for: Ellagitannin HeT obtained from strawberry leaves is oxidized by bacterial membranes and inhibits the respiratory chain
Source: FEBS Open Bio. 2018 Jan 4;8(2):211–8. doi: 10.1002/2211-5463.12361 (PMC5794468; doi:10.1002/2211-5463.12361)
Supplement: Supplementary file 1 — Fig. S1. Oxygen consumption of C. michiganensis suspensions (A 600 = 0.5) treated with water, increasing concentrations of HeT and KCN (5 mm). The values represent average of three repetitions. Error bars represent relative errors. Fig. S2. Infrared spectrum of pure HeT (50 μm). Group assignments correspond to: carbonyls (A: 1737.2) and (B: 1673.3); aromatics (C, 1613.6) and (D, 1518.7); methylenes (E, 1446.7); aromatic esters (F, 1110.3) and (G, 1078.2). Fig. S3. Effect of HeT on the reduction DPPH expressed as percentage of antiradical activity (%AA). Water was used as a control. The values represent means of three repetitions. Error bars represent relative errors. Fig. S4. Differential pulse voltammetry of HeT (0.4 mm) in the oxidative range in aqueous phosphate buffer (pH 7.4). Fig. S5. UV‐visible spectra of HeT with different level of oxidation. Oxidation was achieved by controlled potential electrolysis of HeT (0.4 mm) in phosphate buffer (pH 7.4) at +0.3 V. Spectra were acquired every 30 s. Fig. S6. UV‐visible spectra of (A) bacterial membrane (5 μg·mL−1), (B) pure HeT (0.4 mm), and (C) HeT (0.4 mm) after reacting with the bacterial membrane (5 μg·mL−1). All solutions were prepared in phosphate buffer (pH 7.4). [file FEB4-8-211-s001.pdf]

Supplemental Figures

Supplemental Figure 1. -single columm-

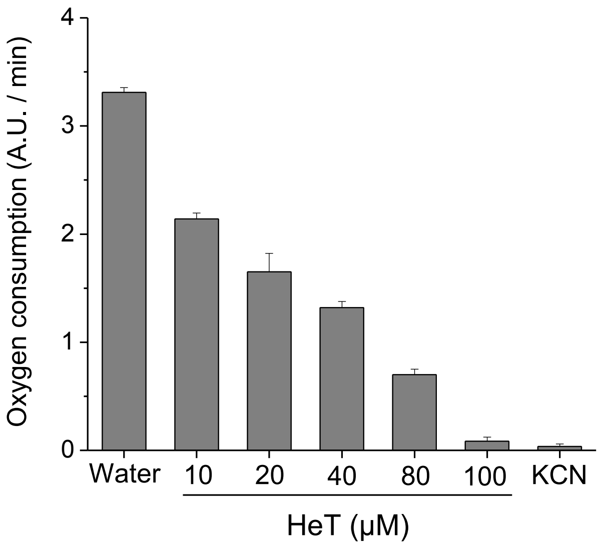

Supplemental Fig.1. Oxygen consumption of *C. michiganensis* suspensions (OD_{600} = 0.5) treated with water, increasing concentrations of HeT and KCN (5 mM). The values represent average of three repetitions. Bars represent relative errors.

Supplemental Figure 2. -single columm-

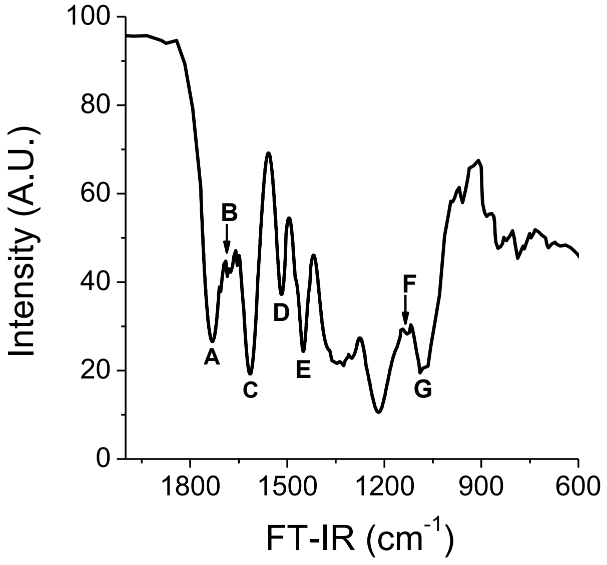

Supplemental Fig.2 Infrared spectrum of pure HeT (50 µM). Group assignments correspond to: carbonyls (A: 1737.2) and (B: 1673.3); aromatics (C, 1613.6) and (D, 1518.7); methylenes (E, 1446.7); aromatic esters (F, 1110.3) and (G, 1078.2).

Supplemental Figure 3. -single columm-

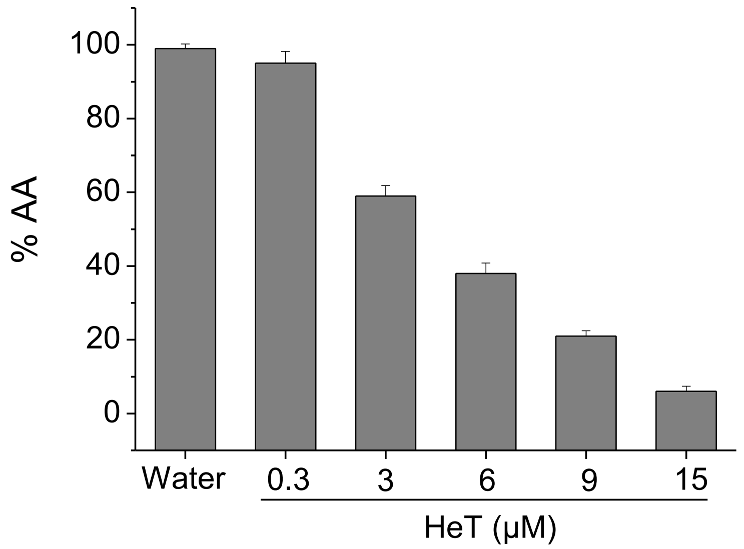

Supplemental Fig. 3. Effect of HeT on the reduction DPPH· expressed as percentage of antiradical activity (%AA). Water was used as a control. The values represent means of three repetitions. Bars represent relative errors.

Supplemental Figure 4. -single columm-

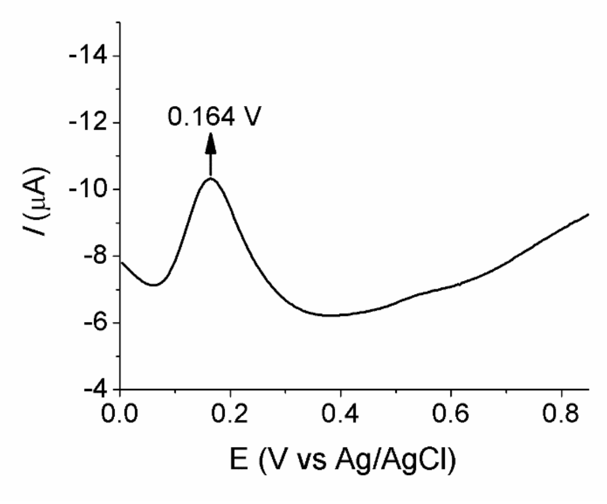

Supplemental Fig. 4. Differential pulse voltammetry of HeT (0.4 mM) in the oxidative range in aqueous phosphate buffer (pH 7.4).

Supplemental Figure 5. -single columm-

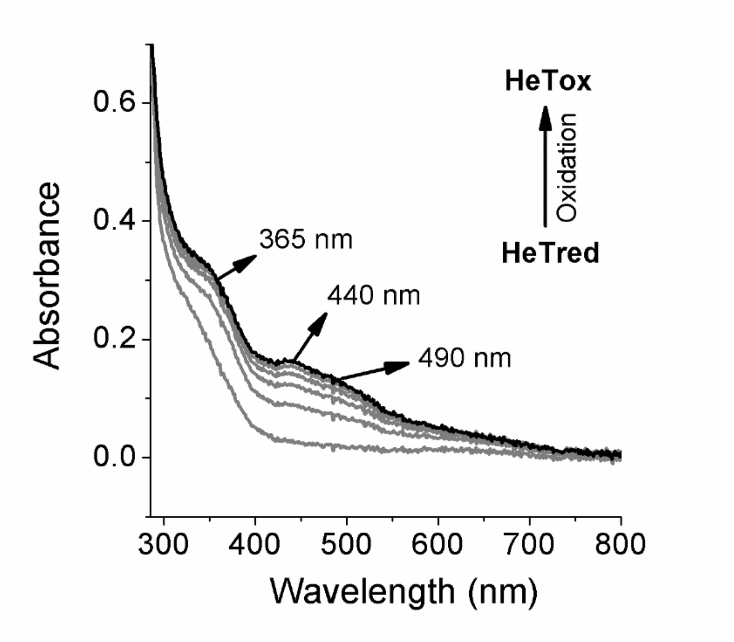

Supplemental Fig. 5. UV-visible spectra of HeT with different level of oxidation. Oxidation was achieved by controlled potential electrolysis of HeT (0.4 mM) in phosphate buffer (pH 7.4) at +0.3 V. Spectra were acquired every 30 s.

Supplemental Figure 6. -single columm-

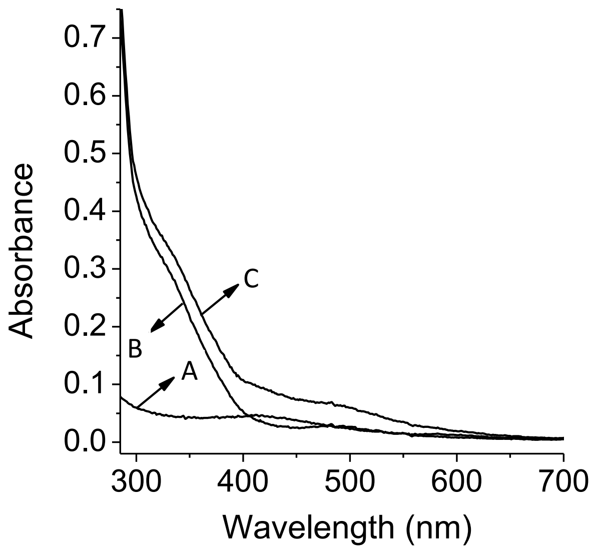

Supplemental Fig. 6. UV-visible spectra of: (A) bacterial membrane (5 µg/mL), (B) pure HeT (0.4 mM), and ~~the differential spectrum between them (C)~~. All solutions were prepared in phosphate buffer (pH 7.4).
